# Survival after Multimodal Treatment Including Surgery for Metastatic Esophageal Cancer: A Systematic Review

**DOI:** 10.3390/cancers14163956

**Published:** 2022-08-16

**Authors:** Thomas Bardol, Lorenzo Ferre, Safa Aouinti, Marie Dupuy, Eric Assenat, Jean-Michel Fabre, Marie-Christine Picot, Regis Souche

**Affiliations:** 1 Department of Digestive Surgery and Transplantation, Montpellier University Hospital, University of Montpellier-Nimes, 641 Avenue du Doyen Gaston Giraud, 34090 Montpellier, France; 2 INSERM, Centre d’Investigation Clinique 1411, Montpellier University Hospital, University of Montpellier-Nimes, 641 Avenue du Doyen Gaston Giraud, 34090 Montpellier, France; 3 Department of Oncology, Montpellier University Hospital, University of Montpellier-Nimes, 641 Avenue du Doyen Gaston Giraud, 34090 Montpellier, France

**Keywords:** esophageal cancer, stage IV, surgery, chemotherapy, radiotherapy, survival

## Abstract

**Simple Summary:**

The management of stage IV esophageal cancer is mostly limited to palliative chemotherapy. In this context, the role and effects of surgery are still controversial. The aim of this systematic review is to assess the survival outcome of surgically treated metastatic esophageal cancer patients. Multimodality treatment, including surgery in curative intent, seems associated with a significant improvement of three years overall survival. Hence, a prospective evaluation of this approach and validation of adequate selection criteria are urgently needed.

**Abstract:**

(1) Background: The management of metastatic esophageal cancer is more often limited to palliative chemotherapy. Limited data are available regarding the role of surgery that remains controversial. The aim of this systematic review is to assess the survival outcome of surgically treated metastatic esophageal cancer patients. (2) Methods: The present systematic review is designed using the PRISMA guidelines and has been registered with PROSPERO (CRD42019140306). Two reviewers independently searched and identified studies dealing with surgery for stage IV esophageal cancer in the Medline and Google Scholar databases between January 2008 and December 2019. (3) Results: Seven retrospective nonrandomized studies, totaling 1756 patients with stage IV esophageal cancer who underwent curative surgery, were included. Our analysis demonstrates a three-year overall survival rate of 23% (CI 95% 17–31) among patients undergoing surgery. Because only two comparative studies were identified, data compilation and relative risk evaluation through meta-analysis were not possible. (4) Conclusions: Multimodality treatment, including surgery in curative intent, seems associated with a significant chance of three-year overall survival. A prospective evaluation of this approach and validation of adequate selection criteria are needed.

## 1. Introduction

Esophageal cancer ranks seventh in terms of incidence with 604,000 new cases worldwide, and is a highly aggressive malignant tumor as it was responsible for one in every 18 cancer deaths in 2020 [1]. Indeed, approximately one out of three patients have a metastatic disease at diagnosis [2]. Survival in patients with stage IV esophageal adenocarcinoma is dramatic, with less than 5% surviving at 5 years [3].

The current guidelines from the National Comprehensive Cancer Network (NCCN) recommend only palliative and supportive care for patients with metastatic esophageal cancer [4,5]. Progress in the treatment of metastatic esophageal cancer remains difficult, unlike for other cancers where metastatic disease could be treated in curative intent in some cases. However, the role of surgery in patients with stage IV esophageal cancer must be continuously reassessed in the light of the developments in preoperative imaging and therapeutic arsenal, both chemotherapy and radiotherapy protocols, which have occurred over the past 15 years.

To date, many retrospective series have reported overall survival data for primary tumor and metastases resected patients, but no prospective randomized trials have been conducted. In addition, the heterogeneity of the patients and the limited number of patients included in each series makes the clinical impact of those studies’ results limited.

To better define the long-term outcomes of multimodal treatments, including surgery in curative intent (esophagectomy +/− surgical excision of metastases) in those patients, we conduct a systematic review of the published studies evaluating surgery in patients with synchronous metastatic esophageal cancer.

## 2. Materials and Methods

### 2.1. Study Design

Data analysis was performed in accordance with PRISMA (Preferred Reporting Items for Systematic Reviews and Meta-Analyses) guidelines [6]. The protocol of this review was recorded in PROSPERO (CRD42019122854).

### 2.2. Research Strategy

We conducted systematic research of the literature available between January 2008 and December 2019. No language restrictions were used. Pubmed and Google Scholar were searched for prospective and retrospective studies, meta-analyses and systematic reviews using different combinations of: (i) the following query for Pubmed (Medline): ((((surgery[Title]) OR (resection[Title])) OR (esophagectomy[Title])) AND ((((metasta*[Title]) OR (stage IV[Title])) OR (stage 4[Title])) OR (M1 disease[Title]))) AND (((((((((Esophageal Neoplasm[Title]) OR (Esophagus Neoplasm[Title])) OR (Esophagus Neoplasms[Title])) OR (Cancer of Esophagus[Title])) OR (Cancer of the Esophagus[Title])) OR (Esophagus Cancer[Title])) OR (Esophagus Cancers[Title])) OR (Esophageal Cancer[Title])) OR (Esophageal Cancers[Title])) or (ii) the following keywords: “esophageal cancer”, “stage IV”, “metastatic”, “cancer”, “esophagus”, and “surgery” in Google Scholar.

### 2.3. Selection Criteria and Outcome Measures

In the case of duplicate publications that reported on similar patient data, only the most recent and complete data sets were considered. Articles were selected in this systematic review according to the follow eligibility criteria:

1. Participants: adults with squamous cell carcinomas (SCCs) or adenocarcinomas (ADKs) with a synchronous metastases of the esophagus; metastatic disease was defined as having a distant metastasis at the time of diagnosis according to the SEER historical stage. The distant stage was defined as a neoplasm that had spread to parts of the body away from the primary tumor through direct extension, discontinuous metastases (e.g., implantation or seeding) to distant organs and tissues, or from the lymphatic system to distant lymph nodes.
2. Intervention: esogastric surgery resection with or without concomitant treatment of metastases.
3. Comparison: patients with stage IV esophageal cancer not undergoing surgery.
4. Outcomes: the main outcome measure was the 3-year overall survival rate. Secondary outcomes were postoperative morbidity and pathological response.

All articles written in the Latin alphabet were considered potentially eligible for inclusion. Case reports, laboratory animal studies and reviews have not been included in this systematic review. All relevant text, tables and figures were reviewed for data extraction.

### 2.4. Quality Assessment of Retrieved Articles

Two authors screened the titles and abstracts of electronically retrieved articles to determine if they were eligible for inclusion. The decision was finalized after reviewing the full text of articles that were relevant to the topic.

The risk of bias and methodological quality of the included cohort studies were explored using the Newcastle–Ottawa scale (NOS) [7].

### 2.5. Data Extraction

The data on country of origin; years of study; study design; characteristics of participants; multimodal treatment, including surgical procedure and outcomes; control of confounding factors; and information on bias as well as available measures of association, including odds and risk ratios, were extracted. Due to the heterogeneous nature of the multimodal treatment, including the surgical procedure and outcomes across the selected studies included in the review, a meta-analysis was not considered appropriate.

### 2.6. Statistical Analyses and Estimation of Risk of Bias

All the analyses were conducted with R (3.6.3 version), packages “meta” and “metafor” (https://cran.r-project.org/mirrors.html, accessed on 1 February 2020). Three-year OS (3yOS) and five-year OS (5yOS) were calculated as the proportion of patients alive at 3 and 5 years, and the total patients included in the study. If not reported, surviving patients were estimated from the survival curves. All statistical measures were considered significant if the *p*-value ≤ 0.05 (i.e., significance level). Heterogeneity between studies was quantified by the between-study variance and the Cochran Q test and/or the I2 statistic, which describes the percentage of variation across studies that is a result of heterogeneity rather than chance [8]. Low, moderate, and high heterogeneities were considered for levels of I2 values of 25–49%, 50–74%, and above 75%, respectively [8]. We used mean difference analysis. The graphical description of the statistical results was illustrated with a forest plot. The evaluation of publication bias was determined by a visual inspection of the funnel plot.

## 3. Results

### 3.1. Study Selection

**Figure 1:**
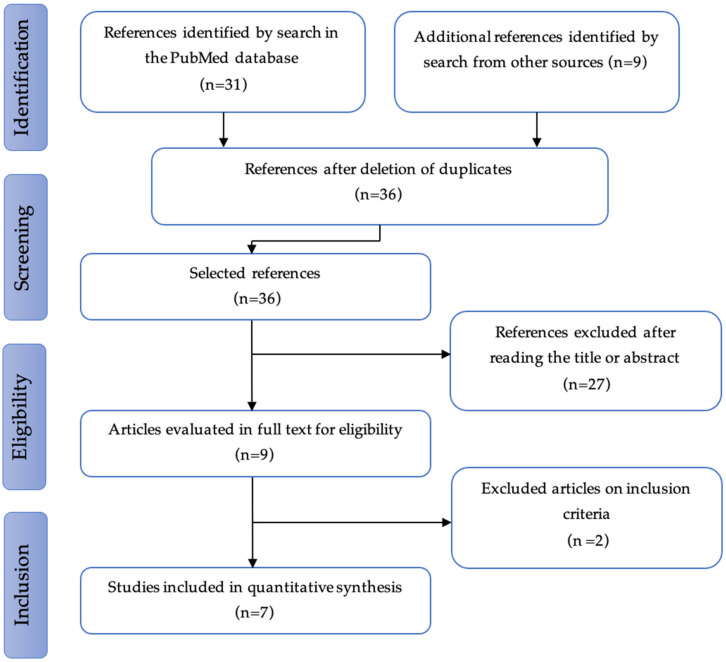
PRISMA flow diagram.

The studies were selected in three consecutive stages: screening, eligibility, and inclusion. Of the 36 non-duplicated references extracted and analyzed, 7 studies, published between 2008 and 2019, were considered as relevant. The results are presented schematically in the PRISMA flow diagram (Figure 1).

### 3.2. Studies and Patients’ Characteristics

Between 2008 and 2019, seven studies fitted the inclusion criteria and were therefore included in the analysis. All the studies were retrospective (level 4) and analyzed separately. Due to the identification of only two comparative studies, data compilation and relative risk assessment performed by meta-analysis were not possible. No randomized trial or meta-analysis were included in the present review. The included studies are described in Table 1.

Only two studies did not report any details on surgical procedures [11,14]. Five studies had a quality score ≥5 and two had a score of 4 assessed using the Newcastle−Ottawa score (Table 2).

A total of 1756 patients undergoing surgery for stage IV esophageal cancer were analyzed. Despite our research strategy that did not include gastric cancer patients, 8.4% of patients had surgery for a cardial or a sub-cardial (AEG II or III) cancer or even a genuine gastric cancer (149/1756 patients). The rate of adenocarcinoma ranged from 64 to 100% of cases. When the metastatic pattern was described, we noticed that 81.5% of patients harbored single-organ metastases disease (198/243 patients). Those metastases were either known before surgery or discovered during surgery [9,12]. Patients’ cancer characteristics are described in Table 3.

#### 3.2.1. Preoperative Multimodal Treatment

Preoperative treatment modalities are summarized in Table 3. In six out of seven studies, patients received a neoadjuvant treatment that relied on chemotherapy, concordant radio-chemotherapy, radiotherapy either alone (induction therapy), or as a consolidation, as presented in the study of Wang et al. The chemotherapy agents used were 5-FU and cisplatin [9], folinic acid protocol, 5-FU + oxaliplatin or cisplatin +/– paclitaxel [10], fluoropyrimidines IV orally alone or in combination with platinum salt and taxane [11], and cisplatin and 5-FU or carboplatin and paclitaxel, or docetaxel, cisplatin, and 5-FU [13]. As far as radiotherapy is concerned, Wang et al. used the three-dimensional conformational method (3DRCT), intensity modulation (IMRT), or proton therapy to deliver the ionizing radiations [11]. Van Daele et al. only reported a total amount of radiation (36 Gy) [13].

#### 3.2.2. Surgical Features and Postoperative Outcomes

The surgical approach was mainly conventional. Indeed, only one minimally invasive procedure was retrieved [12]. Ivor Lewis or trans-hiatal esophagectomies were mostly performed to treat lower-third esophageal cancer. A partial or total gastrectomy was performed to treat cardial, sub-cardial, or gastric cancers. A two-field lymphadenectomy was associated with the case of an esophagectomy. The details on the surgical management of metastases were poorly reported. Schauer et al. treated metastases “if possible” [9]. On the contrary, Blank et al. performed a systematic treatment of secondary lesions as follows partial peritonectomies for localized peritoneal carcinosis, atypical or anatomical hepatic resections for secondary hepatic lesions, and atypical pulmonary resections for secondary pulmonary lesions [10]. Finally, Van Daele et al. reported a complete resection in 11 out of 12 patients reaching a 92% rate of R0 resection [13].

Postoperative morbidity ranged from 25% (5/19 patients) [9] to 51.8% (27/52 patients) [12], and surgery related mortality from 0% (0/12 patients) [13] to 7.7% (4/52 patients) [12]. Unfortunately, three studies did not report any details of surgical postoperative outcomes. The surgical and pathological features are summarized in Table 4.

#### 3.2.3. Pathological Responses

When described, patients harbored a greater proportion of high histological grade tumors (G3 or G4) [3,10,14]. Consequently, the histopathological tumor regression rate following preoperative chemotherapy was low [9,10]. Indeed, only a few patients were considered as good responders (regression grades 1a,b). In Schauer et al.’s study, the pathological response was worse in patients with distant metastases than in the other patients: only 3 out of 19 stage IV patients (15%) showed a good response following chemotherapy, versus 82 of the other 159 patients (52%; *p* = 0.002) [9]. Blank et al. reported that 35 of 159 patients (22%) presented as histological responders [10]. In Saddoughi et al.’s study, the regression rate according to the Becker regression score was not mentioned, but the data on ypTNM status showed that 5 patients were in complete response, i.e., ypT0 (9.5%), 1 patient was ranked ypT1 (2%), 6 patients ypT2 (11.5%), 38 patients ypT3 (73%), and 3 patients ypT4 (4%), confirming the results of the previous studies [12].

### 3.3. Survival Analysis

Median follow-up ranged from 9 to 22 months, while median survival was 12.3 months (median survival reached in 6 out of 7 studies).

**Figure 2:**
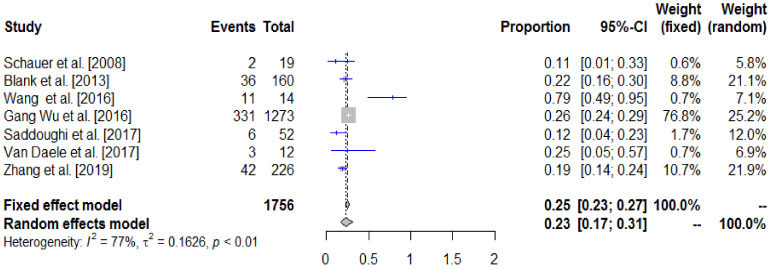
Comparison of 3-year survival rates (forest plot: the gray square represents the individual study effect and its size reflects the weight of the study in the overall analysis). Blue lines represent the confidence intervals of the studies. Studies with no or small squares, which have a lower weight, have greater confidence intervals than studies with large squares. The diamond represents the pooled results of surgery on 3-year survival. The outer edges of the diamond represent the confidence interval of this summary [3,9,10,11,12,13,14].

The overall survival results of the included studies are presented in the forest plot (Figure 2) and summarized in Table 5.

Our analysis showed an overall 3-year survival rate of 23% (CI 95% 17–31) in the operated patients. All studies show a favorable effect on surgery. Heterogeneity I^2^ = 77% was important. By removing the study by Wang et al. [11], heterogeneity decreased to I^2^ = 60%, which could be considered as moderate. This heterogeneity hindered the interpretation of our results. Five-year overall survival rates were reported in five out of seven studies [3,9,10,11,12]. The median 5-y OS was 11%, ranging from 5 to 50%.

#### Survival Prognosis Factors

Blank et al. reported histopathological tumor regression (HR 0.43, 95% CI 0.24–0.74, *p* = 0.002) and category R (R0: HR 0.48, 95% CI 0.28–0.82, *p* = 0.008, R1: HR 0.58, 95% CI 0.35–0.97, *p* = 0.039, R2: reference) as independent prognostic factors [10]. In the study of Wang et al., among the 14 patients that were operated on, the median recurrence-free survival (RFS) was 14.6 months, and the 3- and 5-year OS rates were, respectively, 77% and 50%. However, in the multivariate analysis, the surgery was not statistically associated with a better OS or DFS [11]. In Gang Wu et al.’s study, the median OSs were, respectively, 11 and 15 months for patients with metastatic esophageal cancer that was immediately operated on and for patients undergoing surgery in association with induction or postoperative RCT. Patients with induction radiotherapy had a higher OS rate at 5 years than those with postoperative radiotherapy, 24.7% versus 7.8%, respectively, and a higher median of OS, 20 months versus 12 months, respectively (*p* > 0.0001) [3]. Saddoughi et al.’s multivariate analysis revealed that patients with a high ypT status (ypT3 or T4) had a lower survival rate than those with ypT1 and T2 (HR 4.744, CI95%: 2.001–11.247, *p* = 0.0004) [12].

No correlation was observed between metastatic sites and prognosis [9,10]. Distant metastatic lymph nodes without organ metastasis were associated with improved overall survival compared to organ metastases presented in Wang et al.’s study [11].

### 3.4. Risk of Bias Analysis

**Figure 3:**
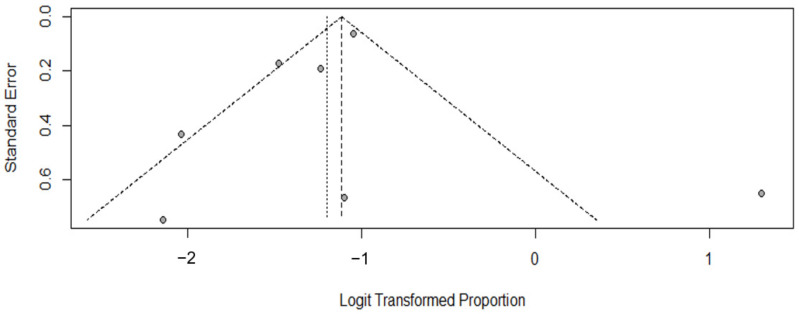
Search for publication bias, funnel graph. Logit transformed proportion represents the odds ratio.

A funnel plot suggests that short studies are more likely to be susceptible to publication bias than lengthier ones, and it is this difference that is assessable [15]. Our funnel plot (Figure 3) is asymmetrical. This unbalanced plot repartition suggests the possibility of publication bias. Finally, three studies were located outside the area under the diagonals confirming the heterogeneity between the analyzed studies.

## 4. Discussion

There are, at present, no standard guidelines for the management of metastatic esophageal cancer patients. To our knowledge, the present study was the first dedicated review of the survival outcomes of primary tumor resection of stage IV esophageal cancer patients. Despite the progress of the treatment of metastatic esophageal cancer, which remains difficult, the place of surgery for patients with metastatic esophageal cancer should be continuously reassessed, given the improvements in the diagnostic and therapeutic armamentarium during the past 15 years. The data available in the literature of these aggressive strategies remain scarce and heterogeneous. However, this is a common situation in current practice since two thirds of esophageal cancers are metastatic. In this study, we demonstrated that a surgical strategy is usually performed in highly selected patients and could be associated with a significant chance of three-year overall survival (23%). The studies mostly address mixed squamous cell carcinomas and adenocarcinomas, synchronous and metachronous metastases, and metastases known preoperatively with metastases discovered intraoperatively. This study also suggested a significant publication bias statistically favoring an aggressive strategy in its patients rather than a non-operative attitude.

As reported in the literature, there are distinct clinical presentations in our current practice: (i) synchronous metastases at diagnosis (including the recent entity of oligometastatic disease) and, more marginally, (ii) metastases diagnosed intraoperatively. Upfront, small metastasis resection (liver, peritoneum, or lung metastases) diagnosed intraoperatively should be avoided and raises the large value of an exhaustive preoperative workup. This workup included a staging laparoscopy for esogastric adenocarcinomas (revealing liver and/or peritoneal metastases in 20% of cases) [16], a positron emission tomography computed tomography (PET/CT), and liver MRI in case of suspicious liver lesions, and furthermore, a CT scan performed shortly before any surgery [17]. On the contrary, some patients with synchronous metastases seemed to benefit from multimodal treatment strategies, including preoperative chemo/radiotherapy, followed by surgical excision in case of response following induction therapy, resulting in intermediate survival outcomes between those of resectable and extensively metastatic esophageal cancer patients [18,19]. Sevedin et al. recently reported that the median OS rates for all esophageal metastatic cancer patients and for those receiving definitive radiation and surgery were, respectively, 6.60 (95% CI 6.51–6.74) and 30.23 months (95% CI 20.47–34.76). An esophagectomy, tumor grades 1–2, and the absence of bone or liver metastases were independently associated with a better survival rate in patients [19].

The question of a curative approach in patients initially metastatic and presenting a complete response following induction chemotherapy is currently debated. We often decide to refer patients to radio-chemotherapy to improve local control. This strategy also enables the patients’ selection in case of the regrowth of distant lesions. When clinical–radiological response/stability is obtained, surgery is sometimes performed on fit patients. Benefit/risk analyses should integrate the potential post-esophagectomy morbidity and mortality of 15–50% and oncologic results that remain uncertain [20]. Indeed, non-metastatic patients with a ypT0N0R0 resection face cancer relapse in 20 to 60% of cases [21]. In this manner, surveillance may lead to better results compared to surgery in terms of survival and quality of life. This concept is valid for non-metastatic patients with resectable squamous cell carcinomas and a randomized controlled study comparing systematic surgery versus surveillance in operable esophageal cancer with a complete clinical responses to radio-chemotherapy (including adenocarcinoma) is ongoing (Esostrate NCT02551458) [22]. The results of this trial will help clinicians in the management of these rare metastatic patients with complete responses.

If there is no consensus on the definition of the “oligometastatic state”, it implies an optimal cancer extension assessment and must include a limited number of metastases and metastatic sites. Oligometastatis is often reported when five or less observable metastatic lesions are present [23,24]. In the present review, only the study conducted by Wang et al. reported the number of metastases (≤1 or >1). Metastatic lesions could be a distant M1 lymph node group (including cervical, mediastinal, gastric, retroperitoneal lymph nodes), bone metastases or visceral metastases, or central nervous system metastases. Satellite lesions in the primary esophageal malignancy, such as skipped esophageal primaries, are usually not considered as metastatic sites.

Oligometastatic cancer includes at least two clinical entities: « true » slowly evolving oligo-metastatic disease and ongoing systemic disease [24]. It is not clear that the first entity is applicable to esophageal cancer. Without biomolecular tools, it is difficult to determine in which entity the patient is. In the case of « false » oligometastatic disease, occult systemic spreading is ongoing, but a limited number of metastatic sites are detectable, and systemic treatment is therefore justified and will need a reassessment of treatment response before considering any surgery. The personalized care plan for potentially oligometastatic patients must consider these challenges to avoid surgery on patients with a partially visible systemic disease or, on the contrary, not to prohibit a surgical strategy for « true » oligometastatic patients with response or stability following induction treatment.

Some limitations should be considered when interpreting the current review. First, only retrospectives studies were included and just two were comparable studies. Moreover, two studies were designed using the SEER database leading to a lack of data. Secondly, the studies were highly heterogenous and the funnel plot confirmed this aspect. The preoperative workup as well as the preoperative treatment greatly differ from one study to another. For example, no laparoscopic exploration of the abdominal cavity was performed prior to the scheduled esophagectomy leading to the diagnosis of metastases during surgery in two studies.

## 5. Conclusions

To conclude, the present review enrolling seven non-randomized retrospective studies and recruiting a total of 1756 patients who underwent surgery for stage IV esophageal cancer, showed an overall three-year survival rate of 23% in the operated patients. Multimodal treatment, including surgery, seems to be associated with a significant chance of overall survival at three years. A prospective trial from M.D. Anderson Cancer Center (ClinicalTrials.gov registration NCT03161522), comparing chemoradiation with or without surgery to systemic therapy alone for esophageal or gastric cancers with oligometastases may provide further insights into the value of aggressively treating metastatic lesions. In Europe, the AIO-FLOT5 trial (RENAISSANCE) is assessing the effect of chemotherapy alone versus chemotherapy followed by surgical resection on survival and quality of life in patients with a limited metastatic adenocarcinoma of the stomach or eso-gastric junction [25]. The first results are expected soon.

The ‘test of time’ in patients with good systemic responses might warrant a more aggressive ablative treatment of oligometastatic disease, even in upper gastrointestinal cancer. However, given the strength of intensified chemo/radiotherapy regimens and the morbidity of surgical procedures, such as an esophagectomy and secondary lesions resection, the treatment of metastatic patients should be tailored and consistently viewed as a quality-of-life approach.

## Figures and Tables

**Table 1:** Summary of included studies.

Study	Design	Country	Study Period	Sample Size	M+ Patients Who Underwent Surgery
Schauer et al., 2008 [9]	Retrospective (vs. M0)	Germany	1996–2006	178	19 (10.7)
Blank et al., 2013 [10]	Retrospective cohort	Germany	1987–2007	707	160 (22.6)
Wang et al., 2016 [11]	Retrospective (vs. no surgery)	U.S.	1999–2012	NR	14 (NA)
Gang Wu et al., 2016 [3]	Retrospective (vs. no surgery)	China	1988–2012	9125	1273 (13.9)
Saddoughi et al., 2017 [12]	Retrospective (known versus i.o. M1)	U.S.	1985–2014	3500	52 (1.5)
Van Daele et al., 2017 [13]	Retrospective cohort	Belgium	2010–2014	602	12 (2)
Zhang et al., 2019 [14]	Retrospective cohort	China	2004–2014	4367	226 (5.2)

Values are numbers (percentages) unless otherwise indicated. NR: not reported; M0: non-metastatic disease; M1 or M+: metastatic disease; i.o.: intra operative; U.S.: United States of America.

**Table 2:** Quality assessment of the selected studies assessed by Newcastle–Ottawa scale (NOS). A study can be awarded a maximum of one star (*) for each numbered item except for the item “comparability of the cohorts based on the design or analysis”. A maximum of two stars (**) can be awarded for “comparability of the cohorts based on the design or analysis”.

Study	Selection				Comparability	Outcome		Score
	Patients Who Underwent Surgery	Representativeness of Exposed Cohort (max = *)	Selection of the Non-Exposed Cohort (max = *)	Ascertainment of Exposure (max = *)	Comparability of the Cohorts Based on the Design or Analysis (max = **)	Assessment of Outcome (max = *)	Adequacy of Follow-up of Cohort (max = *)	
Schauer et al., 2008 [9]	19	*	*	*	*	*	-	5
Blank et al., 2013 [10]	160	*	-	*	-	*	*	4
Wang et al., 2016 [11]	14	*	*	*	*	*	-	5
Gang Wu et al., 2016 [3]	1273	*	*	*	*	*	-	5
Saddoughi et al., 2017 [12]	52	*	*	*	*	*	-	5
Van Daele et al., 2017 [13]	12	*	-	*	-	*	*	4
Zhang et al., 2019 [14]	226	*	*	*	**	*	-	6

**Table 3:** Patients’ cancer characteristics: tumor type, primary and secondary tumor localizations, and preoperative treatment.

			Schauer et al., 2008 [9]	Blank et al., 2013 [10]	Wang et al., 2016 [11]	Gang Wu et al., 2016 [3]	Saddoughi et al., 2017 [12]	Van Daele et al., 2017 [13]	Zhang et al., 2019 [14]
Number of operated patients			19	160	14	1273	52	12	226
Median age (years)			60	NR	59	64	NR	NR	63
Number of adenocarcinomas			19 (100)	160 (100)	13 (93)	815 (64)	46 (89)	9 (75)	169 (74.7)
Primary tumor localization									
	Esophagus								
		Upper third	0	0	0	35 (2.7)	1 (2)	0	6 (2.7)
		Middle Third	0	0	1 (2)	172 (13.5)	5 (10)	ND	26 (11.5)
		Distal Third/AEG I	19 (100)	25 (15.6)	13 (98)	1066 (83.7)	44 (84)	2 (17)	164 (72.6)
	Gastric								
		AEG II or III	0	71 (44.3)	0	0	2 (4)	2 (17)	12 (5.3)
		Other	0	44 (27.5)	0	0	0	0	18 (8.0)
Lymph node metastases			2 (10.5)	16 (10)	11 (79)	NR	NR	NR	150 (66.8)
Metastatic pattern									
	≤ 1 metastasis		NR	NR	7 (50)	NR	NR	NR	NR
	Single-organ metastases		16 (84.2)	119 (68.8)	NR	NR	52 (100)	11 (92)	NR
	Multiple-organ metastases		3 (15.8)	41 (31.2)	NR	NR	0	1 (8)	NR
	Metastatic sites		Lung, liver, distant lymph nodes, bone, peritoneum, spleen, adrenal gland	Peritoneum, lung, liver	Bone, brain, liver, peritoneum, adrenal glands, distant lymph nodes	NR	Lung, liver, peritoneum, distant lymph nodes	Liver, distant lymph nodes, bone	NR
Preoperative treatment									
	Chemotherapy		19 (100)	160 (100)	14 (100)	NR	0	8 (67)	184 (81.4)
	Consolidation RCT		0	0	14 (100)	NR	0	0	0
	Consolidation RT		0	0	0	NR	0	0	0
	RCT		0	0	0	NR	17 (32.6)	4 (33)	NR
	Radiotherapy alone		0	0	0	523 (61.7)	1 (1.9)	0	146 (64.6)

Values are numbers (percentages) unless otherwise indicated. AEG: adenocarcinoma of esogastric junction; NR: not reported; RCT: radio-chemotherapy; RT: radiotherapy.

**Table 4:** Surgical features, postoperative outcomes, and pathological analyses.

	Schauer et al., 2008 [9]	Blank et al., 2013 [10]	Wang et al., 2016 [11]	Gang Wu et al., 2016 [3]	Saddoughi et al., 2017 [12]	Van Daele et al., 2017 [13]	Zhang et al., 2019 [14]
Operated patients	19	160	14	1273	52	12	226
Surgical approach							
Conventional	19 (100)	160 (100)	14 (100)	NR	51 (98)	12 (100)	NR
Laparoscopic	0	0	0	NR	1 (2)	0	NR
Trans-hiatal	NR	20 (13)	NR	NR	5 (10)	0	NR
Two-way (Ivor Lewis)	NR	5 (3)	NR	NR	39 (75)	10 (83)	NR
Three-way (McKeown)	NR	0	NR	NR	3 (5)	0	NR
Other	NR	135 (84)	NR	NR	5 (10)	2 (17)	NR
Metastases treatment	If possible	Systematic	NR	NR	0	11 (92)	NR
Lymphadenectomy	Two-field	Two-field	Two-field	NR	NR	Two-field	NR
Complication rate	5 (25)	56 (35)	NR	NR	27 (51.8)	5 (41.6)	NR
Surgery-related mortality	1 (5)	4 (2.5)	NR	NR	4 (7.7)	0	NR
Pathological features							
R0 resection	NR	66 (41.5)	NR	NR	NR	11 (92)	NR
Tumor regression rate							
Reg 1	3 (15.8)	35 (21.8)	NR	NR	NR	NR	NR
Reg 2	16 (84.2)	124 (77.5)	NR	NR	NR	NR	NR
Histological grade							
G1/2	NR	13 (8)	NR	477 (37.4)	NR	NR	80 (35.4)
G3/4	NR	146 (91)	NR	670 (52.6)	NR	NR	131 (58)

Values are numbers (percentages) unless otherwise indicated. R0: tumor-free resection margin; Reg: regression rate according to Becker regression score; Reg 1: regression rate with less than 50% viable cells; Reg 2: regression rate with more than 50% viable tumor cells; G: histological grading system; NR: not reported.

**Table 5:** Oncological follow-up and survival outcomes.

Study	Patients Who Underwent Surgery	Median Follow-Up (Months)	Mortality Rate (Percentage)	Median Survival (Months)	1-Year OS (Percentage)	3-Year OS (Percentage)	5-Year OS (Percentage)
Schauer et al., 2008 [9]	19	10	74	9	32	10	5
Blank et al., 2013 [10]	160	20.9	2.5 (30 days)	13.6	NR	22.8	11
Wang et al., 2016 [11]	14	NR	NR	Not reached	NR	77	50
Gang Wu et al., 2016 [3]	1273	NR	NR	15	76	26	17.5
Saddoughi et al., 2017 [12]	52	10.6	NR	10.8	29	12	6
Van Daele et al., 2017 [13]	12	22	NR	22	41	28	NR
Zhang et al., 2019 [14]	226	9	14 (90 days)	11	45	18.7	NR

Values are numbers unless otherwise indicated; NR: not reported.

## Data Availability

The data presented in this study are available on request from the corresponding author.

## References

[1] Sung H., Ferlay J., Siegel R.L., Laversanne M., Soerjomataram I., Jemal A., Bray F. (2021). Global Cancer Statistics 2020: GLOBOCAN Estimates of Incidence and Mortality Worldwide for 36 Cancers in 185 Countries. CA Cancer J. Clin..

[2] Horner M. SEER Cancer Statistics Review, 1975–006. http://seer.cancer.gov/csr/1975_2006.

[3] Wu S.-G., Xie W.-H., Zhang Z.-Q., Sun J.-Y., Li F.-Y., Lin H.-X., Bao Y., He Z.-Y. (2016). Surgery combined with radiotherapy improved survival in metastatic esophageal cancer in a surveillance epidemiology and end results population-based study. Sci. Rep..

[4] Ajani J.A., D’Amico T.A., Almhanna K., Bentrem D.J., Besh S., Chao J., Das P., Denlinger C., Fanta P., Fuchs C.S. (2015). Esophageal and esophagogastric junction cancers, version 1.2015. J. Natl. Compr. Cancer Netw..

[5] Ichida H., Imamura H., Yoshimoto J., Sugo H., Kajiyama Y., Tsurumaru M., Suzuki K., Ishizaki Y., Kawasaki S. (2013). Pattern of postoperative recurrence and hepatic and/or pulmonary resection for liver and/or lung metastases from esophageal carcinoma. World J. Surg..

[6] Page M.J., McKenzie J.E., Bossuyt P.M., Boutron I., Hoffmann T.C., Mulrow C.D., Shamseer L., Tetzlaff J.M., Akl E.A., Brennan S.E. (2021). The PRISMA 2020 statement: An updated guideline for reporting systematic reviews. Syst. Rev..

[7] Lo C.K.-L., Mertz D., Loeb M. (2014). Newcastle-Ottawa Scale: Comparing reviewers’ to authors’ assessments. BMC Med. Res. Methodol..

[8] Higgins J.P., Thompson S.G., Deeks J.J., Altman D.G. (2003). Measuring inconsistency in meta-analyses. Bmj.

[9] Schauer M., Stein H., Lordick F., Feith M., Theisen J., Siewert J.R. (2008). Results of a multimodal therapy in patients with stage IV Barrett’s adenocarcinoma. World J. Surg..

[10] Blank S., Lordick F., Dobritz M., Grenacher L., Burian M., Langer R., Roth W., Schaible A., Becker K., Bläker H. (2013). A reliable risk score for stage IV esophagogastric cancer. Eur. J. Surg. Oncol..

[11] Wang J., Suri J.S., Allen P.K., Liao Z., Komaki R., Ho L., Hofstetter W.L., Lin S.H. (2016). Factors predictive of improved outcomes with multimodality local therapy after palliative chemotherapy for stage IV esophageal cancer. Am. J. Clin. Oncol..

[12] Saddoughi S.A., Reinersman J.M., Zhukov Y.O., Taswell J., Mara K., Harmsen S.W., Blackmon S.H., Cassivi S.D., Nichols F., Shen K.R. (2017). Survival after surgical resection of stage IV esophageal cancer. Ann. Thorac. Surg..

[13] Van Daele E., Scuderi V., Pape E., Van de Putte D., Varin O., Van Nieuwenhove Y., Ceelen W., Troisi R., Pattyn P. (2018). Long-term survival after multimodality therapy including surgery for metastatic esophageal cancer. Acta Chir. Belg..

[14] Zhang R., Zou J., Li P., Li Q., Qiao Y., Han J., Huang K., Ruan P., Lin H., Song Q. (2020). Surgery to the primary tumor is associated with improved survival of patients with metastatic esophageal cancer: Propensity score-matched analyses of a large retrospective cohort. Dis. Esophagus.

[15] Sedgwick P. (2013). Meta-analyses: How to read a funnel plot. Bmj.

[16] De Graaf G., Ayantunde A., Parsons S., Duffy J., Welch N. (2007). The role of staging laparoscopy in oesophagogastric cancers. Eur. J. Surg. Oncol..

[17] Mariette C., Piessen G., Triboulet J.-P. (2007). Therapeutic strategies in oesophageal carcinoma: Role of surgery and other modalities. Lancet Oncol..

[18] Carmona-Bayonas A., Jiménez-Fonseca P., Echavarria I., Cánovas M.S., Aguado G., Gallego J., Custodio A., Hernández R., Viudez A., Cano J.M. (2018). Surgery for metastases for esophageal-gastric cancer in the real world: Data from the AGAMENON national registry. Eur. J. Surg. Oncol..

[19] Seyedin S.N., Parekh K.R., Ginader T., Caster J.M. (2021). The Role of Definitive Radiation and Surgery in Metastatic Esophageal Cancer: An NCDB Investigation. Ann. Thorac. Surg..

[20] Takeda F.R., Cecconello I. (2021). The complex assessment of anastomosis’ perfusion following esophagectomy: Set in stone?. Eur. J. Surg. Oncol..

[21] Blum Murphy M., Xiao L., Patel V.R., Maru D.M., Correa A.M., Amlashi F.G., Liao Z., Komaki R., Lin S.H., Skinner H.D. (2017). Pathological complete response in patients with esophageal cancer after the trimodality approach: The association with baseline variables and survival—The University of Texas MD Anderson Cancer Center experience. Cancer.

[22] Bedenne L., Mariette C. Comparison of Systematic Surgery Versus Surveillance and Rescue Surgery in Operable Oesophageal Cancer with a Complete Clinical Response to Radiochemotherapy (Esostrate). Identifier: NCT02551458. NCT02551458.

[23] Weichselbaum R.R., Hellman S. (2011). Oligometastases revisited. Nat. Rev. Clin. Oncol..

[24] Hellman S., Weichselbaum R.R. (1995). Oligometastases. J. Clin. Oncol..

[25] Al-Batran S.-E., Goetze T.O., Mueller D.W., Vogel A., Winkler M., Lorenzen S., Novotny A., Pauligk C., Homann N., Jungbluth T. (2017). The RENAISSANCE (AIO-FLOT5) trial: Effect of chemotherapy alone vs. chemotherapy followed by surgical resection on survival and quality of life in patients with limited-metastatic adenocarcinoma of the stomach or esophagogastric junction—A phase III trial of the German AIO/CAO-V/CAOGI. BMC Cancer.

